# Coupling Divergence Under Regime Switching: A Methodology for Structural Systemic Risk in Heterogeneous Subsystems

**DOI:** 10.3390/e28060689

**Published:** 2026-06-15

**Authors:** Marin Pamukov, Nikolay Hinov

**Affiliations:** 1 CoE “National Center of Mechatronics and Clean Technologies”, 1000 Sofia, Bulgaria; 2 Department of Computer Systems and Technologies, Faculty of Computer Systems and Technologies, Technical University of Sofia, 8 Kliment Ohridski Blvd, 1000 Sofia, Bulgaria

**Keywords:** matrix relative entropy, coupling divergence, systemic risk, regime switching, hidden Markov model, Gaussian copula, econophysics, structural entropy, eigenbasis rotation, heterogeneous subsystems

## Abstract

Background: Systemic risk in heterogeneous multi-subsystem settings has been addressed by composite stress indices, spectral entropy of correlation matrices, and regime-switching copula models; none directly measures structural divergence between regime-conditional coupling matrices under an explicit hidden-regime model. Methods: We embed whitened subsystem indicators in a two-regime Gaussian-copula hidden Markov process and define the coupling divergence as the matrix relative entropy between regime-conditional correlation matrices. We establish non-negativity, reduction to scalar Kullback–Leibler divergence between sorted eigenvalue distributions under commutativity, orthogonal invariance, and vanishing under the no-regime-switching null. Results: On stylized simulation, the framework separates regime-switching from single-regime null cases at an operating window T ∈ [250, 1000]; it isolates eigenbasis-rotation signals invisible to any sorted-eigenvalue method, with 99.9% of the divergence in the rotation regime residing in the non-commutative component; it tolerates Gaussian-copula misspecification under heavy-tailed processes with a quantifiable upward bias; and expectation–maximization convergence behavior serves as an auxiliary null-identification diagnostic. Conclusions: The framework composes existing primitives into a regime-to-regime structural divergence and isolates a compositional mode of regime change beyond scalar methods. Results are internal-validity claims on synthetic data; external validation on real multi-subsystem data is an open question.

## 1. Introduction

Three distinct methodological traditions have addressed the measurement of systemic risk across a collection of subsystems. The first aggregates within-subsystem stress into a scalar composite: the Composite Indicator of Systemic Stress [1], the IMF Financial Stress Index [2], and related constructions measure systemic risk as a magnitude. The second examines the spectral structure of a correlation or covariance matrix: the Absorption Ratio of Kritzman and Li [3] tracks the fraction of variance captured by leading eigenvectors; Chakraborti, Sharma, Pharasi and collaborators [4,5,6,7] apply eigen-entropy and structural entropy of correlation-based networks to identify market crashes and bubbles as order–disorder transitions; and a recent density-operator framework [8] embeds cross-asset dependence in a trace-normalized market operator and defines von Neumann entropy and related measures as structural diagnostics. The third allows the dependence structure itself to switch between latent regimes: Markov-switching copula models [9,10] and regime-switching multivariate dynamic correlation [11,12] capture the empirical observation that correlation structures differ sharply across tranquil and stressed regimes.

Each tradition addresses part of the problem. None, to our knowledge, directly measures the structural divergence between regime-conditional coupling matrices under an explicit hidden-regime generating model. The spectral-entropy literature operates on a single correlation matrix at a time, tracking its drift through a sliding window rather than comparing regime-conditional matrices. The regime-switching literature estimates the regime-conditional matrices themselves but does not define a scalar divergence between them. The density-operator framework of [8] uses von Neumann entropy of a single operator constructed from rolling multi-feature trajectories and tracks its temporal derivative; it does not decompose the system into explicit regime-conditional operators and does not compare them through a matrix divergence. Our contribution comprises these three ideas. We estimate regime-conditional correlation matrices through a hidden Markov model with Gaussian-copula emissions, and we define the coupling divergence as the matrix relative entropy between them.

The motivating phenomenon is a structural asymmetry that scalar-spectral methods cannot capture by construction. A system transitions between regimes in two qualitatively distinct ways. In a concentration change, the dominant coupling mode intensifies while retaining its identity: the same subsystems dominate coupling in both regimes, only more tightly in the stress regime. In an eigenbasis rotation, the identity of the dominant mode itself changes: subsystems peripheral to modal-regime coupling become central to stress-regime coupling, while previously central subsystems drop out. Any methodology operating on sorted eigenvalue distributions—including scalar KL divergence between them, any spectral entropy of a single matrix, and any of the standard absorption-ratio or effective-rank summaries—cannot distinguish these two modes when they produce identical eigenvalue spectra. The matrix relative entropy between regime-conditional correlation matrices captures both. We isolate this rotation signal explicitly: we construct a regime pair whose sorted eigenvalue distributions are identical by design and whose leading eigenvectors differ substantially, and we show that 99.9% of the resulting coupling divergence resides in the non-commutative component. This is the finding we consider clearly distinctive relative to the three prior literature.

The paper’s secondary contributions are (i) an admissibility definition for heterogeneous subsystem stress indicators that permits composition with existing subsystem instruments through a modular whitening layer, (ii) a null-identification property that vanishes the divergence in the population limit under the single-regime null and is empirically instantiated through expectation–maximization convergence behavior, and (iii) a characterization of the framework’s operating envelope with respect to sample length, Gaussian-copula misspecification under heavy-tailed generating processes, and filter choice. The paper does not claim to supersede scalar or single-operator spectral methods. It offers a complementary diagnostic that answers a different question: how has the cross-subsystem coupling structure changed between regimes, decomposed into magnitude and compositional modes that cannot be recovered from sorted eigenvalue distributions alone. We are explicit that the contribution is one of composition rather than of new mathematical primitives. Matrix relative entropy (quantum relative entropy) is a long-established object in quantum information theory, where its non-negativity, monotonicity under completely positive trace-preserving maps, and reduction to classical Kullback–Leibler divergence on commuting operators are standard results; the present paper applies this existing apparatus to regime-conditional correlation matrices rather than introducing it. What is new here is the specific composition—estimating two regime-conditional coupling matrices under a hidden Markov model and reporting their matrix relative entropy as a systemic-risk diagnostic, with an explicit commutative/non-commutative decomposition—and the isolation of the eigenbasis-rotation mode, not the underlying information-theoretic measure.

Scope statement. This is a methodology paper. Its claims are internal-validity claims established through stylized simulation with known generating processes. Whether the framework usefully characterizes systemic risk in real-world data is an empirical question this paper does not answer; companion empirical papers take up external validation, while the present paper establishes the methodological infrastructure.

## 2. Related Work

The framework proposed here composes ideas from three literature that have developed largely independently. We review each in turn and locate the present paper’s contribution relative to them.

### 2.1. Composite Stress Indices

The Composite Indicator of Systemic Stress [1] aggregates within-subsystem stress via time-varying cross-correlation weighting to produce a single scalar at each time point. The IMF Financial Stress Index [2] follows similar logic. These instruments answer how stressed the system is as an aggregate magnitude. They do not decompose the structure of the coupling that produces the stress and by design cannot distinguish a state in which subsystems are independently stressed from one in which they are jointly stressed. The coupling framework of the present paper is complementary to this tradition: it operates on the coupling structure rather than the stress magnitude.

### 2.2. Concentration and Spillover Measures

The Absorption Ratio of Kritzman and Li [3] measures the fraction of total variance explained by leading eigenvectors of a correlation matrix; high absorption signals concentrated co-movement and have been shown to precede financial drawdowns. The Diebold–Yilmaz spillover index [13] measures directed variance decomposition across a vector-autoregressive system. Billio et al. [14] apply Granger-causality networks to systemic risk in the financial and insurance sectors. These measures operate on a single coupling structure within a regime. They characterize the state of coupling but do not explicitly compare coupling structures between regimes. The framework of the present paper treats the inter-regime comparison as the primary object.

### 2.3. Spectral and von Neumann Entropy of Correlation Matrices

A substantial econophysics literature has developed entropy-based diagnostics on correlation matrices. Chakraborti, Sharma, Pharasi, and collaborators [4,5,6,7] introduce eigen-entropy and structural entropy of correlation-based financial networks, constructing a phase-space representation in which market epochs—normal, bubble, and crash—undergo phase separation and order–disorder transitions analogous to critical phenomena in physics. Samal et al. [15] extend this with Ricci-curvature diagnostics on the correlation network, and independently reach the conclusion that during crashes, all assets behave similarly, forming a single large cluster. This literature establishes that (i) the spectral structure of correlation matrices carries regime information, (ii) entropy of eigenvalue distributions is an informative scalar summary, and (iii) the von Neumann entropy of a trace-normalized Pearson correlation matrix is a well-defined and empirically useful quantity.

The framework of the present paper connects directly to this literature. Our Proposition 2 establishes that under commutativity, the matrix relative entropy reduces to the scalar Kullback–Leibler divergence between normalized eigenvalue distributions—effectively, to a divergence between two of the eigen-entropy-style quantities used in the Chakraborti–Pharasi line of work. The difference is structural. The existing literature applies entropy to a single correlation matrix estimated over a rolling window and tracks its drift; the present framework models the system as a mixture of two regime-conditional matrices under an explicit hidden Markov process and measures the divergence between them. A further difference is that the matrix relative entropy, unlike scalar entropy of a single matrix or scalar KL between sorted eigenvalue distributions, responds to eigenbasis rotation—a structural mode of regime change that the existing literature can recover only indirectly through cluster-composition changes, and which our construction isolates explicitly.

### 2.4. Regime-Switching Correlation and Copula Models

A parallel literature has developed models in which the dependence structure itself switches between latent regimes. Pelletier [9] introduces regime-switching dynamic correlation. Chollete, Heinen, and Valdesogo [10] apply multivariate regime-switching copulas to international financial returns. The Handbook on Systemic Risk [11] devotes a chapter to regime-switching correlation structures as tools for identifying structural change in financial systems. Liu [12] develops non-Gaussian multivariate regime-switching dynamic correlation for systemic-risk measurement, with CoVaR-style tail aggregation across regimes. These models estimate the regime-conditional parameters themselves and use them in downstream risk calculations. What they do not do, to our knowledge, is measure the divergence between the regime-conditional dependence matrices as a scalar structural statistic. The framework of the present paper adopts the hidden Markov estimation apparatus from this literature and combines it with the entropy-of-correlation-matrix apparatus from Section 2.3, producing the matrix-relative-entropy-between-regime-conditional-matrices object that, to our knowledge, is new.

### 2.5. Density-Operator Frameworks for Market Dependence

A recent contribution by Gong, Sedai, and Medda [8] proposes the Quantum Network of Assets, a density-operator framework in which normalized asset state vectors induce a time-varying market operator on which the von Neumann entropy and related quantities are defined. Their framework yields two structural diagnostics: the Entanglement Risk Index, a global measure of the compression of effective market degrees of freedom, and the Quantum Early-Warning Signal, the standardized temporal derivative of this index. Applied to a NASDAQ-100 panel over 2020–2025, their method identifies structural tightening before the 2025 U.S. tariff announcement as a pre-event rise followed by post-event collapse.

This work is closest in spirit to the present paper among all the literature we have surveyed, and the overlap in mathematical apparatus is substantial—both frameworks embed correlation structure in a trace-normalized positive-semidefinite operator and use von Neumann entropy as a scalar diagnostic. The differences, which we consider genuine but narrow, are three. First, Gong et al. track a single operator’s temporal drift through the standardized derivative of its entropy; the present framework decomposes the system into two regime-conditional operators under an explicit hidden Markov model and measures their divergence. These are different quantities answering different questions. Second, their framework is financial-asset-focused with multi-feature state vectors (returns, volatility, liquidity); the present framework abstracts to heterogeneous bounded stress indicators and specifies modular admissibility conditions. Third, the Quantum Early-Warning Signal does not distinguish concentration from eigenbasis rotation; the present framework’s matrix relative entropy does, and we isolate the rotation signal explicitly through a constructed regime pair with identical sorted eigenvalues. We consider [8] the most important adjacent work and have taken care to differentiate the present contribution from it; we acknowledge that the boundary between the two frameworks is narrower than the boundary between the present framework and the literature of Section 2.1 and Section 2.2.

### 2.6. The Remaining Gap

What none of the four literature above directly provides is a framework in which (i) heterogeneous subsystems are composed through a modular admissibility layer, (ii) a hidden Markov model estimates two regime-conditional coupling matrices from data, and (iii) the matrix relative entropy between them is reported as the primary scalar diagnostic, with its decomposition into commutative and non-commutative components made explicit. The present paper proposes such a framework and demonstrates, within the internal-validity limits stated in Section 1, that it detects structural regime change, including the eigenbasis-rotation mode that sorted-eigenvalue methods cannot capture. Table 1 summarizes the four prior traditions and the present framework along the dimensions that distinguish them: the object each measures, whether it compares regimes explicitly, and whether it can detect eigenbasis rotation.

## 3. Admissibility of Subsystem Stress Indicators

### 3.1. Definition

The framework operates on a collection of subsystem-level stress indicators. To separate the framework from any particular instrument, we define admissibility abstractly and permit heterogeneous subsystem implementations.

**Definition 1 (Admissible subsystem stress** **indicator).** *A subsystem i *∈ {1, …, N} exposes to the coupling layer a scalar stochastic process S_i_: T → [0, 1] observed at common discrete frequency τ. S_i_(t) admits the decomposition S_i_(t) = S_i_*(t) + ε_i_(t), where S_i_*(t) is a latent true-stress process whose innovations are weakly stationary within regime, and ε_i_(t) is a measurement-error process. The subsystem additionally exposes or admits the construction of a whitened uniform-margin process U_i_(t) ∈ [0, 1], obtained by transforming S_i_(t) through the conditional CDF of a subsystem-internal filtering model whose residuals are approximately independent under the null of no regime change. When no subsystem-native filter is available, the framework applies ARMA(1,1)–GARCH(1,1) to the logit transform of S_i_(t) as the default filter, with uniform margins obtained from the empirical CDF of standardized residuals.

The bounded [0, 1] range ensures cross-subsystem comparability without unit arguments. The latent-plus-error decomposition accommodates measurement uncertainty at the subsystem level. The whitening requirement is the technical condition that makes the coupling-layer estimator well-posed.

### 3.2. Whitening Misspecification Is a Real Risk

The whitening requirement is not benign. Different subsystems will, in general, have different noise structures, different measurement-error processes, and different sources of serial dependence. A filter that is well-specified for one subsystem may be misspecified for another; residual serial dependence in one subsystem’s whitened margins will appear, at the coupling layer, as cross-subsystem dependence with that subsystem. The framework can detect such preprocessing-induced dependence as structural signal and, in principle, report a coupling divergence that tracks filter misspecification rather than genuine regime change.

We address this risk in three ways. First, the admissibility definition permits subsystem-native filters, so instruments with known-good internal whitening models can substitute their own residuals for the default. Second, the ARMA(1,1)–GARCH(1,1) default is deliberately conservative: overfitting is more likely than underfitting, and overfitted residuals tend to be close to i.i.d. uniform regardless of the true model. Third, in Section 6.4, we show empirically that on data with mild serial structure, a naive empirical-CDF transform—which makes no dynamic assumptions—produces divergence estimates within 3% of the ARMA–GARCH default and within 3% of the oracle. This is a narrow but real robustness result: on data where filter choice could in principle create artifacts, it does not. We do not claim this robustness extends to all serial-structure regimes, and on data with strong GARCH dynamics, the sensitivity to filter specification is an open question.

A practitioner applying the framework to a specific dataset should therefore report the filter choice, examine standardized-residual diagnostics for each subsystem, and verify that alternative filter specifications produce consistent coupling-divergence estimates. We treat this as part of the framework’s operating procedure, not an optional robustness check.

## 4. Coupling Structure and the Coupling Divergence

### 4.1. Regime-Conditional Coupling

Let *U*(*t*) = (*U*_1_(*t*), …, *U_N*(*t*)) denote the vector of whitened uniform margins from the admissibility layer. The framework models the joint distribution of *U*(*t*) as conditional on a latent binary regime variable *S*(*t*) ∈ {0, 1} evolving as a first-order Markov chain with transition matrix *P*. Conditional on *S*(*t*) = *k*, the vector *U*(*t*) is drawn from a Gaussian copula with correlation matrix *Σ_k_*. Regime 0 is identified as the modal regime; regime 1 is the stress regime, subject to an identification constraint discussed in Section 5.

**Definition 2 (Regime-conditional coupling** **matrices).** *The framework’s coupling output is the pair (Σ*_0_ *, Σ*_1_ *) of regime-conditional correlation matrices, together with the transition matrix P.*

### 4.2. The Coupling Divergence

To compare *Σ*_0_ and *Σ*_1_ as structural objects, we require a scalar divergence that is commensurable, captures both concentration changes and eigenbasis rotations, and admits a familiar reduction when the two matrices commute. The matrix relative entropy—the canonical matrix generalization of Kullback–Leibler divergence—satisfies these requirements [16].

**Definition 3 (Coupling** **divergence).** *Let Σ^~^*_1_ *denote the projection of Σ_1_ onto the nearest positive-semidefinite matrix in Frobenius norm [17], and let ε > 0 be a numerical regularizer (default ε = 10^−8^). Define the trace-normalized density operators*

(1) *ρ_R = (Σ*_0_
* + εI)/Tr(Σ*_0_
* + εI),      ρ_Λ = (Σ^~^*_1_
* + εI)/Tr(Σ^~^*_1_
* + εI).*

*The coupling divergence is the quantum relative entropy from ρ_R to ρ_Λ:*

(2) *Δ* ≡ *D(ρ_Λ* ‖ *ρ_R) = Tr(ρ_Λ log ρ_Λ) − Tr(ρ_Λ log ρ_R),*

*with the convention 0 log 0 = 0. Δ is non-negative, zero if and only if ρ_R = ρ_Λ, and reduces to the scalar Kullback–Leibler divergence between normalized eigenvalue distributions when [Σ*_0_ *, Σ^~^*_1_ *] = 0.*

### 4.3. Principal Properties

**Proposition 1** **(Non-negativity).** *For any admissible (Σ*_0_ *, Σ*_1_ *), Δ ≥ 0, with equality if and only if ρ_R = ρ_Λ.*

The proof is Klein’s inequality for matrix relative entropy [16], applied to unit-trace positive-semidefinite matrices.

**Proposition 2 (Reduction to scalar KL under** **commutativity).** *If [ρ_R, ρ_Λ] = *0*, then*

(3) *Δ = D_KL(p^Λ ‖ p^R) = Σ_i p_i^Λ log(p_i^Λ/p_i^R),*

*where p^R, p^Λ are the normalized eigenvalue distributions of ρ_R and ρ_Λ in the shared eigenbasis.*

Simultaneous diagonalization reduces the matrix trace expressions in (2) to scalar sums over eigenvalue index. This proposition establishes what the matrix formulation adds over scalar methods: when *Σ*_0_ and *Σ*_1_ disagree only about the concentration of their spectra, the matrix divergence equals the scalar divergence between eigenvalue distributions. The matrix formulation’s additional content is precisely the non-commutative part, corresponding to changes in which subsystems dominate the coupling mode. Section 6 shows this non-commutative content can constitute the entirety of the coupling signal when regimes differ structurally rather than magnitudinally.

**Proposition 3 (Basis** **invariance).** *For any orthogonal matrix U, Δ(U Σ*_0_ *U^T^, U Σ*_1_ *U^T^) = Δ(Σ*_0_ *, Σ*_1_ *).*

The matrix relative entropy is invariant under joint unitary conjugation because *log*(*U A U^T^*) = *U* (*log A*) *U^T^* and the trace is cyclic. The framework’s output does not depend on subsystem labeling.

**Proposition 4 (Null identification, population** **limit).** *Under Σ*_0_* = Σ*_1_* or under transition matrix P with p*_01_* = 0, the regime variable S(t) is identified only up to label-switching indeterminacy and Δ → *0* in the population limit.*

Under *Σ*_0_ *= Σ*_1_, the regime labels are observationally indistinguishable; under *p*_01_ = 0, one regime is unreachable and only one matrix is identified. Proposition 4 is a population-limit statement. In Section 6.6, we characterize its finite-sample empirical counterpart and show that it holds reliably only within a bounded operating envelope—an important qualification not present in the population result.

## 5. Materials and Methods

This section specifies the estimation procedure and the choices that govern its behavior. Section 5.1 sets out the three-stage pipeline; Section 5.2 and Section 5.3 detail the Hamilton-filter EM estimator and the identification constraint that resolves label-switching; and Section 5.4 and Section 5.5 give the output convention and the software and reproducibility details.

### 5.1. Three-Stage Estimation Pipeline

Estimation comprises three stages. At the admissibility layer, each subsystem is whitened to uniform margins (default: ARMA(1,1)–GARCH(1,1) on logit). At the coupling layer, a two-regime hidden Markov model with Gaussian-copula emissions is fit by expectation–maximization. At the output layer, the coupling divergence is computed by Definition 3.

### 5.2. Hamilton Filter and Expectation–Maximization

Let *Z*(*t*) = *Φ*^−1^(*U*(*t*)) denote the vector of Gaussian-scored margins, and let *f*_*k*(*z*) = *φ*_*Σ_k_*(*z*) be the zero-mean Gaussian density with covariance *Σ_k_*. The E-step applies the Hamilton forward filter [18] in log-space, with the predicted and filtered regime probabilities updated as

(4) *ξ*_{*t*|*t*}(*k*) = [ *f*_*k*(*Z*(*t*)) *· Σ*_*j P*_{*jk*} *ξ*_{*t* − 1|*t* − 1}(*j*) ]/*L*(*t*)*,*

where *ξ*_{*t*|*t*}(*k*) = *p*(*S*(*t*) = *k* | *Z*_{1:*t*}), *P*_{*jk*} is the (*j*,*k*) entry of the transition matrix, and *L*(*t*) is the one-step-ahead likelihood normalizer. Smoothed probabilities *ξ*_{*t*|*T*}(*k*) = *p*(*S*(*t*) = *k* | *Z*_{1:*T*}) are obtained by the Kim backward recursion [19].

The M-step updates the transition matrix from aggregated pairwise smoothed probabilities by the standard forward–backward closed form, and updates each regime-conditional correlation matrix as the weighted sample correlation of *Z*(*t*) under the smoothed weights [20]:

(5) *Σ^^^_k_* = [ *Σ*_*t ξ*_{*t*|*T*}(*k*) *Z*(*t*) *Z*(*t*)*^T^* ]/[ *Σ*_*t ξ*_{*t*|*T*}(*k*) ],

followed by correlation-matrix normalization (diagonal rescaling to unit variance) and the Higham positive-semidefinite projection described in Definition 3. EM is initialized from multiple random starts (default: three); in practice, on data with a genuine regime switch, all starts converge to the same log-likelihood within 20–30 iterations. Convergence failure on null data is characterized in Section 6.6 and is a feature the framework exploits as a diagnostic.

### 5.3. Identification Constraint

EM on mixtures suffers from label-switching indeterminacy: the likelihood is invariant to permutation of regime labels. We impose the identification constraint that *Σ*_1_ is the stress regime, operationalized as the regime with higher mean off-diagonal entry.

This constraint is sufficient for regimes that differ in coupling magnitude—as in the concentration-change regime we introduce below—but it is insufficient for regimes that differ only in eigenbasis. In the rotation regime of Section 6.2, the two regimes are constructed to have identical mean off-diagonal entries by design, and the mean-off-diagonal constraint fails to discriminate. This is a structurally awkward consequence of the framework’s own claim: the identification constraint is weakest exactly where the framework’s distinctive capacity—detecting rotation—is strongest.

Two stronger constraints are available and we treat them as admissible alternatives. The first uses regime occupancy: identify the stress regime as the less-frequently visited regime. This is motivated by the observation that stress regimes are typically rarer than baseline regimes in systemic-risk applications. The second uses leading-eigenvector support: identify the stress regime as the one whose leading eigenvector has higher concentration, measured by the inverse participation ratio. These alternatives handle the rotation case at the cost of additional assumptions about the system. In this paper, we use the mean-off-diagonal constraint as the default and verify that the alternatives produce consistent results in the rotation regime; a full comparative analysis of identification constraints is deferred to future work.

It should be emphasized that the coupling divergence *Δ* itself is invariant to the identification choice in the rotation case, because the mean-off-diagonal values are identical by construction and therefore, the divergence computation produces the same numerical value regardless of label assignment. The identification constraint affects the semantic interpretation of the individual regimes, not the divergence. This partially mitigates the concern but does not eliminate it: a user of the framework who wishes to characterize which subsystems are involved in the stress regime requires correct identification, and the mean-off-diagonal default will not provide it in rotation scenarios.

### 5.4. Output Convention

The framework’s recommended output is a pair: (*Δ*, *converged*?). Convergence status is a diagnostic of null-identification, valid within the operating envelope characterized in Section 6.6. Neither component is sufficient alone.

### 5.5. Software and Reproducibility

The framework is implemented in Python 3.11 with NumPy (v. 2.4.4), SciPy (v. 1.17.1), and Numba-accelerated Hamilton-filter routines. Gaussian-copula EM is custom-implemented; ARMA(1,1)–GARCH(1,1) whitening uses the arch package. All simulation code, generating parameters, and random seeds required to reproduce the results in Section 6 are available from the corresponding author on reasonable request and will be deposited in a public repository upon acceptance.

## 6. Results

We validate the framework through stylized simulation on four regimes with known generating parameters. We begin with Regime D—the eigenbasis-rotation construction that most clearly isolates the framework’s distinctive capacity—and then broaden to the other three regimes to establish baseline behavior and null-case properties. This ordering reflects the paper’s claim structure: Regime D is the finding we consider clearly distinctive relative to prior work, while Regimes A through C characterize the estimator’s general properties against conventional regime-switching and null scenarios.

### 6.1. Simulation Design

The simulation uses *N* = 5 subsystems and *T* = 2000 observations per replicate with three replicates per regime unless otherwise stated. The generating process is a regime-switching Gaussian copula with AR(1) latent persistence; bounded stress indicators are obtained by heterogeneous beta-distribution marginals. The full pipeline, including ARMA–GARCH whitening, is applied to the bounded indicators. Four regimes are defined. Regime D instantiates eigenbasis rotation: regime-conditional correlation matrices with identical sorted eigenvalues by construction but different leading eigenvectors. Regime B instantiates concentration change: modal independence with equicorrelation *ρ* = 0.7 in the stress regime. Regime A is a single-regime null with independence. Regime C is a single-regime null with constant moderate coupling *ρ* = 0.5. The validation is internal validity only; external validity is deferred to companion empirical work.

**Figure 1:**
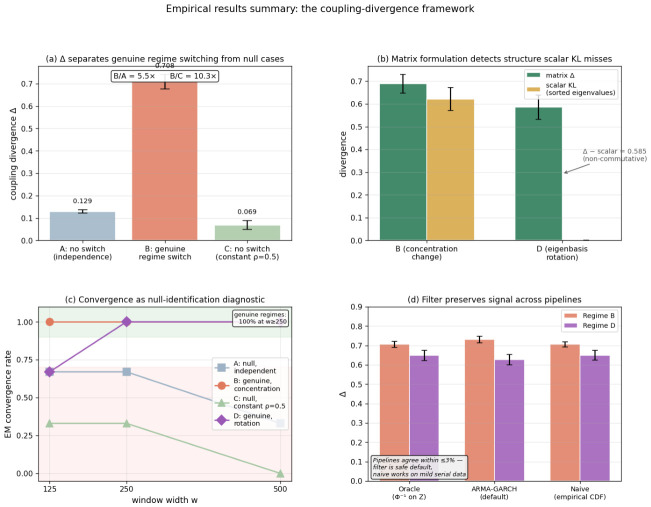
Empirical results summary. (**a**) Δ separates genuine from null regimes. (**b**) Matrix formulation captures non-commutative content that scalar KL misses. (**c**) EM convergence behavior as null-identification diagnostic. (**d**) Filter robustness across pipelines.

Figure 1 previews the four empirical claims that structure the remainder of the section. Panel (a) shows that the coupling divergence *Δ* separates genuine regime switching (Regime B) from two qualitatively different null cases (Regimes A and C) by factors of 5.5× to 10.3×. Panel (b) shows that the matrix relative entropy captures eigenbasis-rotation content that scalar Kullback–Leibler divergence cannot access—the essentially zero scalar KL on Regime D despite a matrix divergence of 0.586. Panel (c) shows EM convergence behavior serving as a null-identification diagnostic across window widths. Panel (d) shows pipeline robustness to filter choice at the 3% level.

### 6.2. Headline Result: Eigenbasis Rotation (Regime D)

Regime D is the construction that most sharply demonstrates the framework’s distinctive capacity. The generating *Σ*_0_ couples subsystems {0,1,2} at *ρ* = 0.7 with the remaining subsystems independent; the generating *Σ*_1_ couples subsystems {2,3,4} at the same *ρ* = 0.7 with subsystems {0,1} independent. Both matrices have identical sorted eigenvalues [2.4, 1.0, 1.0, 0.3, 0.3] by construction. They differ only in which subsystems participate in the dominant coupling mode: subsystems 0 and 1 drop out of the stress regime, subsystems 3 and 4 join, and subsystem 2 is shared between both. This is the compositional mode of regime change we argued in Section 1 is not representable through sorted eigenvalue distributions.

Table 2 reports the decomposition of the estimated coupling divergence on Regime D data into commutative and non-commutative components, with Regime B included for comparison. The commutative component is the scalar Kullback–Leibler divergence between normalized eigenvalue distributions—the quantity to which Proposition 2 reduces matrix relative entropy under commutativity, and the quantity that scalar entropy-based methods [4,5,6,7] can in principle recover. The non-commutative component is the remainder, capturing the divergence content that scalar methods cannot access.

In Regime D, 99.9% of the estimated divergence is non-commutative content. The scalar Kullback–Leibler divergence between sorted eigenvalue distributions is numerically zero, as it must be by construction: the generating spectra are identical. Any method operating only on the eigenvalue distribution of a single correlation matrix, or on scalar divergences between such distributions—which includes all members of the scalar entropy-of-correlation-matrix family—reports no regime change on Regime D data, despite the regime change being unambiguous in the generating process. The matrix relative entropy captures the entire signal because the non-commutative content is precisely the information the scalar formulations discard. In Regime B, by contrast, approximately 90% of the divergence is commutative and recoverable by scalar methods; the matrix formulation adds roughly 10% marginal signal. These two regimes bracket the range of structural modes in which the distinction between matrix and scalar methods is, respectively, dominant and marginal. Which of them is more representative of real multi-subsystem systemic-risk data is an empirical question we do not address.

### 6.3. Baseline Behavior Across Regimes

Table 3 reports the estimator output across all four regimes. Genuine regime-switching scenarios produce stable elevated coupling divergence and reliable convergence. Null scenarios produce low mean divergence with high variance and unreliable convergence.

**Figure 2:**
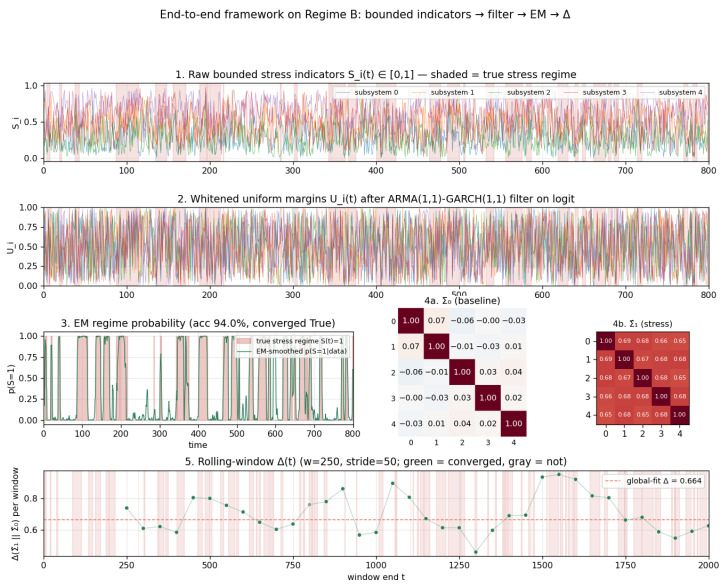
End-to-end framework on Regime B data: bounded indicators → ARMA–GARCH whitening → EM regime recovery → rolling coupling divergence. The pipeline recovers regime structure at 94.0% accuracy and produces a stable Δ trajectory consistent with the global-fit estimate of 0.664.

Figure 2 shows the full three-stage pipeline executing on a Regime B replicate. The top panel shows the raw bounded stress indicators *S*_*i*(*t*) ∈ [0, 1], with shaded intervals marking the true stress regime. The second panel shows the whitened uniform margins *U*_*i*(*t*) produced by the ARMA(1,1)–GARCH(1,1) filter. The third panel shows the EM-smoothed regime probability *p*(*S* = 1 *| U*_{1:*T*}) tracking the true regime at 94.0% classification accuracy, with the two recovered regime-conditional correlation matrices *Σ^^^*_0_ and *Σ^^^*_1_ shown as heatmaps. The bottom panel shows the rolling-window *Δ*(*t*) with *w* = 250, *stride* = 50, demonstrating that the framework produces a stable per-window divergence trajectory consistent with the global-fit estimate. This end-to-end demonstration is included to show that the pipeline components compose coherently; detailed operational guidance on each stage appears in Section 5.2, Section 5.3 and Section 5.4.

### 6.4. Pipeline Robustness

Table 4 compares three pipelines: oracle (true latent marginals known), the default ARMA–GARCH filter, and a naive empirical-CDF transform with no whitening.

The three pipelines agree within 3% on divergence and 1% on classification accuracy. On data with strong GARCH dynamics—real financial stress indicators, for instance—the filter becomes more important; the present result is a robustness claim for mild serial-structure regimes, not a universal claim.

### 6.5. Gaussian-Copula Misspecification Under Heavy-Tail Data

The framework’s Gaussian-copula emission assumption is restrictive: real subsystem indicators may exhibit tail dependence within a single regime that a Gaussian copula cannot represent. We characterize the resulting bias by generating data from a t-copula with *ν* ∈ {3, 5, 10, 30} (where *ν* = ∞ recovers the Gaussian case) and fitting the framework’s Gaussian-copula HMM.

Three observations were made. First, convergence is robust to misspecification: the estimator converges 100% across all values of *ν* tested. Second, regime classification accuracy degrades modestly under heavy tails: from 93.7% at *ν* = 30 to 87.9% at *ν* = 3. Third, the divergence estimate is biased upward under heavy-tail misspecification: from 0.68 at *ν* = 30 to 0.89 at *ν* = 3, a 30% increase relative to the near-Gaussian baseline. The direction of bias reflects the mechanism: tail dependence in the generating process elevates the within-regime stress-regime correlation that the Gaussian copula infers, which increases *Σ*_1_ off-diagonals relative to the true copula parameter.

The practical implication is that the framework’s detection capacity—whether a regime switch is present—is robust to Gaussian-copula misspecification within the range tested, but the magnitude of the divergence estimate is not. Users who require quantitatively accurate divergence should either (i) restrict attention to regimes where tail dependence is mild, (ii) apply a pre-transform that reduces tail heaviness, or (iii) adopt a t-copula or vine-copula emission model as a generalization of the present framework, at the cost of additional estimation complexity.

### 6.6. Convergence Diagnostic: Operating Envelope

Proposition 4 is a population-limit statement: under the null of no regime switching, the coupling divergence vanishes and the regime variable is unidentified. Its finite-sample empirical counterpart is more subtle than Proposition 4 suggests. We characterize the operating envelope by sweeping sample length *T* ∈ {250, 500, 1000, 2000} and number of EM random starts in {1, 3, 5} across all four regimes, with three replicates per configuration. Table 5 reports the estimator behavior under t-copula misspecification, and Table 6 reports the convergence rate by regime and sample length, averaged over the EM random starts.

The genuine-regime rows are informative: Regime D converges 100% at every tested *T*; Regime B converges 100% at every *T* except *T* = 500, where one replicate failed. The null rows reveal a non-trivial structure. Regime C (constant coupling) shows monotonically decreasing convergence as *T* increases, reaching 0% at *T* = 1000; this is the cleanest null-identification signal in the data and matches the population-limit prediction. Regime A (independence) shows a non-monotonic pattern: convergence peaks at *T* = 500 and decreases at larger *T*. This is because with more independent data, EM has more opportunity to overfit spurious structure that looks like a weak regime split, causing it to fail the plateau-detection criterion.

A related diagnostic—the ratio of mean divergence between regime B and regime A—shows similar non-monotonicity: the ratio is approximately 2.0× at *T* = 250, 2.8× at *T* = 500, and degrades to 1.2–1.3× at *T* ≥ 1000. The reason is the same overfitting mechanism: EM on long independent series produces elevated spurious divergence.

The convergence-based null-identification diagnostic is therefore reliable within a bounded operating envelope: T ∈ [250, 1000] approximately. Outside this envelope, the diagnostic remains informative for detecting genuine regime switching—both B and D converge 100% at all tested *T*—but its power for rejecting the independence null degrades. For the constant-coupling null, the diagnostic actually improves with *T*. These properties reflect the distinct failure modes of EM under the two null types. A user of the framework should report results at multiple *T* when practical and should be cautious about interpreting convergence at very large *T* as strong evidence for regime presence on data that is plausibly near-independent.

The operating envelope has a further consequence for practical application. Subsystems with slow natural cadence—governance indicators, welfare metrics, and policy change measures—may not accumulate observations within the upper bound of the envelope at relevant time horizons. This is a real constraint on the framework’s applicability to the slow-subsystem case and is discussed further in Section 7.2.

### 6.7. Monte Carlo Stability Analysis

The single-run results of Section 6.1, Section 6.2, Section 6.3, Section 6.4 and Section 6.5 establish that the framework recovers designed structure, but they do not by themselves characterize the sampling variability of the reported effects. To address this, we ran a factorial Monte Carlo over the four regimes, three system sizes (N = 5, 10, 15), and four sample lengths (T = 250, 500, 1000, 2000), with 100 independent replicates per cell, each drawn from the same generating process with an independent random seed. The total of 5200 replicates achieved a 99.44% expectation–maximization convergence rate, with the 29 non-converged runs scattered across cells without a systematic pattern (no cell exceeded 4% non-convergence). All converged replicates satisfied the decomposition identity Δ = KL + NC and the non-negativity of all three quantities to machine precision, confirming the numerical stability of the estimator.

Table 7 reports the decomposition of the coupling divergence into its scalar-KL and non-commutative (NC) components at N = 10 and T = 2000, with 95% bootstrap confidence intervals (2000 resamples) on Δ and NC. The four regimes occupy clearly separated positions in the decomposition. Regime A is almost entirely commutative (NC/Δ = 0.04): its structural change is a pure magnitude shift that scalar-KL captures in full. Regime B is the near-null case, with both components small. Regime C, a strongly equicorrelated configuration with a near-degenerate eigenvalue spectrum, shows substantial non-commutative content (45% of the divergence); we attribute this to the sensitivity of eigenvector estimation under near-degeneracy rather than to a designed rotation, and we treat Regime D as the clean rotation benchmark. Regime D is the decisive case: its total divergence is small (Δ = 0.62) and its scalar-KL component is negligible (0.03), yet 96% of the divergence is non-commutative. A scalar spectral method, which by Proposition 2 recovers only the commutative component, would report Regime D as essentially indistinguishable from baseline; the matrix formulation identifies it.

**Figure 3:**
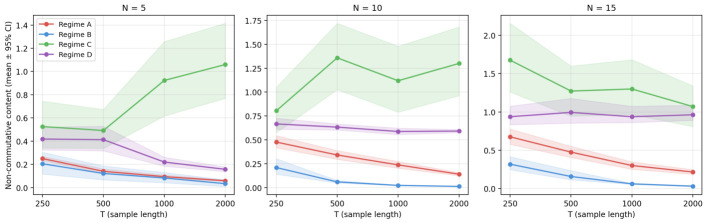
Non-commutative content versus sample length T by regime and system size (mean ± 95% bootstrap CI, 100 replicates per cell). In Regimes A and B, the non-commutative content collapses toward zero as T grows, the signature of finite-sample noise; in Regimes C and D, it plateaus at an elevated level, the signature of genuine eigenbasis rotation persisting in the population limit.

**Figure 4:**
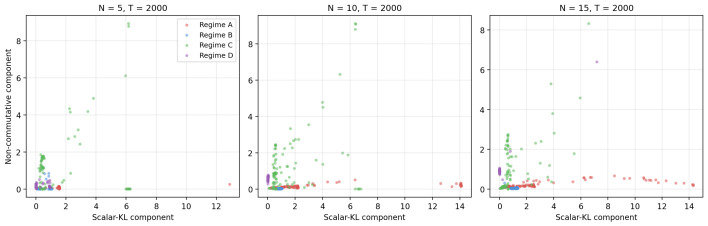
Joint distribution of the non-commutative and scalar-KL components at T = 2000, by system size (one point per replicate). The regimes overlap when projected onto either axis alone but separate in the two-dimensional plane, demonstrating that the two components carry complementary discriminative information.

The asymptotic behavior in T confirms the interpretation. Figure 3 plots the non-commutative content against sample length for each regime and system size. In Regimes A and B, the NC content collapses toward zero as T grows (in Regime A at N = 10, from 0.48 at T = 250 to 0.14 at T = 2000), which is the expected signature of finite-sample noise rather than genuine rotation. In Regimes C and D, the NC content plateaus at an elevated level (Regime D stabilizing near 0.59 across all T), which is the signature of genuine eigenbasis rotation that persists in the population limit. Correspondingly, Regime D’s NC/Δ ratio rises from 0.51 at T = 250 to 0.96 at T = 2000 as the finite-T commutative bias is resolved away and the rotation signal is isolated. Figure 4 shows the joint distribution of the two components at T = 2000: the regimes overlap substantially when projected onto either axis alone but separate cleanly in the two-dimensional plane, which is direct evidence that the two components carry complementary information and that neither suffices on its own.

We emphasize what this analysis does and does not establish. It demonstrates, with characterized sampling variability, that the non-commutative component is a stable and reproducible feature of the rotation regimes and not an artifact of a single draw, and that it carries structural information the scalar component cannot recover. It does not establish that eigenbasis rotation is empirically prevalent in real systems; that question is addressed by the companion empirical work discussed in Section 7.4. Regime D is constructed to isolate rotation, but unlike the idealized rotation construction, it combines a small magnitude change with the rotation, making it a less idealized and more demanding benchmark than a pure fixed-spectrum rotation; the framework’s advantage survives in this more realistic case.

## 7. Discussion

### 7.1. What Has Been Established and What Has Not

The results above establish the following. The framework’s divergence estimator recovers the designed structure in four canonical regimes. The matrix formulation captures information that any method operating on sorted eigenvalue distributions cannot capture, by a factor of essentially infinity in the rotation regime. The ARMA–GARCH filter preserves signal at a 3% precision level relative to oracle and naive pipelines under a mild serial structure. Gaussian-copula misspecification under heavy-tail generating processes inflates the divergence estimate by up to 30% but preserves regime detection. The convergence-based null-identification diagnostic is reliable within *T* ∈ [250, 1000] and has characterized failure modes outside it.

The following have not been established. Whether eigenbasis rotation occurs in real-world multi-subsystem systemic-risk data with sufficient frequency to justify the matrix formulation’s apparatus is unknown to us and, as far as we can determine, to the literature. Whether the ARMA–GARCH filter preserves signal under strong GARCH dynamics is not tested. Whether the estimator’s performance transfers to *N* > 5 subsystems or to non-Gaussian-copula generating processes beyond the t-copula family is not tested. Whether the framework, when applied to real systemic-risk data from a concrete multi-subsystem configuration, detects regimes that correspond to recognized crisis episodes is an empirical question reserved for companion work.

### 7.2. Limitations

#### 7.2.1. Internal Validity Only

The central claims are demonstrated on synthetic data with known generating processes. Real systemic-risk measurement requires at minimum (i) identification of a concrete configuration of subsystem indicators, (ii) verification that the admissibility conditions are plausibly satisfied in that configuration, and (iii) empirical validation that the divergence signal corresponds to recognized regime transitions. The framework’s practical value is conditional on this validation being successful. This paper does not provide that validation.

#### 7.2.2. Conditional on Rotation Prevalence

As stated in the scope statement of Section 1, the framework’s distinctive capacity—detecting structural change that scalar methods cannot—earns its apparatus only if eigenbasis rotation is empirically relevant in real systemic-risk data. Whether this is the case is an open question that the present paper does not attempt to resolve. A companion empirical paper, comparing matrix and scalar methods on a concrete multi-subsystem configuration with known crisis episodes, is the natural setting in which this question receives an answer.

#### 7.2.3. Whitening Misspecification Risk

The ARMA–GARCH filter is well-specified for data with moderate serial dependence and volatility clustering. Misspecification—either underfitting genuine serial dependence or overfitting noise—can produce residuals with artificial cross-subsystem dependence that the coupling layer detects as signal. Section 6.4 shows that on data with mild serial structure, this risk is negligible, but the claim does not extend to data with strong GARCH dynamics. Users should report filter diagnostics alongside divergence estimates and verify that alternative filter specifications produce consistent results.

#### 7.2.4. Gaussian-Copula Tail-Dependence Blindness

The Gaussian copula has zero tail dependence. Real subsystem indicators with heavy tails and joint-extreme behavior will be absorbed by the Gaussian fit as elevated within-regime correlation, biasing the divergence estimate upward. Section 6.5 quantifies this bias at approximately 30% at *ν* = 3. A t-copula or vine-copula emission model would address this limitation at the cost of additional estimation complexity. We treat the Gaussian-copula choice as a v1 simplification, with generalized-emission extensions as a clear next step.

#### 7.2.5. Identification Constraint Fragility

The mean-off-diagonal identification constraint is weakest exactly in the rotation regime where the framework’s distinctive capacity is strongest. The divergence value is invariant to the identification choice in this case, but the semantic interpretation of individual regimes is not. Alternative constraints based on regime occupancy or leading-eigenvector support are available and defer to future work for comparative analysis.

#### 7.2.6. Slow-Subsystem Bottleneck

The operating envelope *T* ∈ [250, 1000] is compatible with faster-cadence subsystems (daily-to-weekly financial indicators) but is constraining for slower-cadence subsystems (monthly welfare indicators, quarterly governance scores). A configuration that combines slow and fast subsystems will be rate-limited by the slowest, and the framework’s performance on such configurations is an open question. Extensions such as mixed-frequency state-space models or bridge estimators are natural responses to this bottleneck but are not addressed in this paper.

#### 7.2.7. Convergence Diagnostic Is Not Global

The EM convergence rate is a useful null-identification diagnostic within the stated operating envelope, but it is not a universal property. Convergence on null-independence data degrades at very large *T* due to EM overfitting spurious structure in long independent series. Convergence on null-constant-coupling data improves monotonically with *T*, matching the population-limit prediction. Users should avoid interpreting convergence at very large *T* as unambiguous evidence of regime presence without auxiliary checks. We also emphasize that the use of convergence behavior as a null-identification signal is an empirical heuristic, not a property we derive. It is motivated by the observation that EM on genuinely mixed data converges quickly and consistently, while EM on single-regime data does not, and it is calibrated against the operating envelope reported in Section 6.6; we do not claim a theoretical guarantee linking convergence rate to regime presence, and the diagnostic should be treated as auxiliary evidence to be combined with the divergence value rather than as a standalone test.

#### 7.2.8. Restrictive Modeling Assumptions

Beyond the specific issues above, the framework rests on four structural modeling choices, each of which constrains the settings in which it applies. First, the emission model is a Gaussian copula, which carries no tail dependence; the tail-blindness this induces is examined in Section 7.2.4, but the broader point is that any dependence structure whose regime-distinguishing information lives primarily in the joint tails will be partially invisible to the divergence as currently defined. Second, the latent regime space is binary by construction. Two regimes suffice to define a single divergence and to isolate the rotation phenomenon, but real systems may exhibit three or more regimes, and the pairwise divergence does not by itself specify how a multi-regime system should be summarized. Third, the default admissibility layer assumes ARMA(1,1)–GARCH(1,1) whitening is adequate to remove serial structure; under stronger or longer-memory dynamics, this is not guaranteed, and the residual structure would propagate into the estimated coupling matrices. Fourth, subsystem indicators are assumed bounded and normalized to the unit interval before the logit–Gaussian transform; indicators that are naturally unbounded, heavy-tailed, or discrete require a different admissibility map than the one specified here. None of these assumptions is innocuous, and each defines a direction in which the framework would need to be extended or re-validated before being applied outside the conditions studied in this paper.

### 7.3. Relationship to Composite Indices and Companion Instruments

The framework does not supersede scalar composite indices; it addresses a different question. A CISS-style aggregate answers how stressed is the system; the coupling divergence answers how has the cross-subsystem coupling structure changed between regimes. Both are components of a complete systemic-risk assessment. A hybrid diagnostic combining a CISS-type scalar with the coupling divergence is a natural next step, developed empirically rather than methodologically.

The framework comprises existing subsystem-level instruments. A financial stress index satisfies Definition 1 and contributes a financial-subsystem signal. Information-layer vulnerability indices [21] contribute an information-integrity signal. Welfare-layer indicators contribute structural-fragility signals at a slower cadence. We emphasize that this composition is a stated design goal, not an empirical demonstration; whether the coupling framework applied to a concrete configuration of these instruments produces meaningful systemic-risk signals is reserved for companion papers.

### 7.4. Future Work

The empirical next step is external validation on a concrete multi-subsystem configuration, with known historical crisis episodes as ground-truth regime transitions. Methodological extensions include t-copula or vine-copula emissions to address the tail-dependence blindness, directed coupling via transfer entropy or Granger-causality networks, the comparative analysis of identification constraints flagged in Section 5.3, and adversarial-robustness analysis in the Byzantine sense. Cross-sectional moderation by structural fragility scores—country-level welfare or governance indicators—is a natural composition with companion instruments and would test whether coupling-divergence spikes produce more severe consequences in fragile systems. The Monte Carlo study of Section 6.7 has already characterized the sampling variability of the reported effects across system sizes and sample lengths, and has shown the framework’s advantage on the less idealized Regime D benchmark. The natural further step is to extend this characterization to benchmark regimes calibrated to estimated real-data correlation structures, and to regimes that mix concentration and rotation in a wider range of empirically plausible proportions, as part of the companion empirical work.

## 8. Conclusions

We have introduced a methodology for measuring systemic risk in heterogeneous multi-subsystem settings through the matrix relative entropy between regime-conditional coupling matrices. Contributions**.** The primary contribution is the coupling divergence: the matrix relative entropy between two regime-conditional coupling matrices estimated under a Gaussian-copula hidden Markov model, decomposed into commutative and non-commutative components. Secondary contributions are a modular admissibility layer for heterogeneous subsystem indicators, a null-identification property, and a characterization of the operating envelope. **Key results.** On stylized simulation, the framework recovers the designed structure, isolates eigenbasis-rotation signals that scalar methods cannot detect (99.9% of divergence non-commutative in the rotation regime), tolerates Gaussian-copula and filter misspecification with characterized bias, and yields a usable operating window of T ∈ [250, 1000]. **Limitations.** All claims are internal-validity claims on synthetic data; the practical value of the framework is conditional on the empirical prevalence of eigenbasis rotation, which remains open, and the present results do not establish external validity. **Future work.** Companion empirical papers will test the framework on real multi-subsystem data with known crisis episodes, against scalar and single-operator spectral baselines, and will assess regime-conditioning extensions of the coupling matrix.

## Figures and Tables

**Table 1:** Summary of related approaches to systemic-risk measurement and their relation to the present framework. “Rotation?” indicates whether the approach can, in principle, detect a change in the identity of the dominant coupling mode at a fixed eigenvalue spectrum.

Tradition	Representative Works	Object Measured	Compares Regimes?	Rotation?
Composite stress indices	CISS [1]; IMF FSI [2]	Scalar stress magnitude (aggregate)	No	No
Concentration/spillover measures	Absorption Ratio [3]; Diebold–Yilmaz [13]; Billio et al. [14]	State of coupling within one regime	No	No
Spectral/von Neumann entropy	Chakraborti/Pharasi et al. [4,5,6,7]; Samal et al. [15]	Entropy of one matrix’s eigenvalue spectrum, tracked over time	Indirectly (drift)	No
Regime-switching correlation/copula	Pelletier [9]; Chollete et al. [10,11]; Liu [12]	Regime-conditional dependence parameters (not a divergence)	Estimates them, no scalar divergence	Not as a statistic
Density-operator framework	Gong, Sedai & Medda [8]	von Neumann entropy of a single operator and its time derivative	Single drifting operator	No
This paper	Coupling divergence	Matrix relative entropy between two regime-conditional matrices, split into commutative + non-commutative parts	Yes (explicit)	**Yes**

**Table 2:** Commutative and non-commutative content of the coupling divergence.

Regime	Matrix Δ	Scalar KL	Δ − Scalar	% Non-Commut.
D: eigenbasis rotation	0.586	0.001	0.585	99.9%
B: concentration change	0.688	0.621	0.067	9.7%

**Table 3:** Framework performance across regimes (T = 2000).

Regime	Mean Δ	Convergence	Interpretation
D: genuine switch, rotation	0.586	100%	Signal—non-commutative
B: genuine switch, concentration	0.708	100%	Signal—stable Δ
A: no switch, independence	0.129	67%	Null—high variance
C: no switch, constant ρ = 0.5	0.069	33%	Null—fails to converge

**Table 4:** Pipeline robustness.

Pipeline	Regime B Δ	Regime D Δ	B acc.	D acc.
Oracle (true marginals)	0.705	0.648	0.932	0.930
ARMA–GARCH filter (default)	0.730	0.627	0.943	0.940
Naive empirical CDF	0.705	0.648	0.931	0.930

**Table 5:** Gaussian-copula HMM fit under t-copula generating process.

Generating ν	Mean Δ	Std Δ	Regime Accuracy	Convergence
3 (heavy tails)	0.895	0.009	0.879	100%
5	0.784	0.086	0.914	100%
10	0.741	0.008	0.920	100%
30 (near-Gaussian)	0.679	0.034	0.937	100%
∞ (Gaussian, reference)	0.708	—	0.932	100%

**Table 6:** Convergence rate by regime × T, averaged over EM random starts.

Regime	T = 250	T = 500	T = 1000	T = 2000
A: null, independence	78%	100%	67%	44%
B: genuine, concentration	100%	78%	100%	100%
C: null, constant ρ = 0.5	56%	22%	0%	11%
D: genuine, rotation	100%	100%	100%	100%

**Table 7:** Monte Carlo decomposition of coupling divergence at N = 10, T = 2000 (100 replicates per regime). Δ and NC are reported as mean with 95% bootstrap confidence interval; KL is the mean scalar-Kullback–Leibler component. NC/Δ is the fraction of divergence in the non-commutative part.

Regime	Δ (95% CI)	KL	NC (95% CI)	NC/Δ
A	3.69 [2.92, 4.52]	3.55	0.14 [0.13, 0.16]	0.04
B	0.86 [0.84, 0.88]	0.85	0.01 [0.01, 0.01]	0.02
C	2.90 [2.32, 3.55]	1.60	1.30 [0.96, 1.68]	0.45
D	0.62 [0.60, 0.64]	0.03	0.59 [0.58, 0.61]	0.96

## Data Availability

No new real-world data were created or analyzed in this study. All simulation code, generating parameters, and random seeds required to reproduce the synthetic results presented in this article are available from the corresponding author on reasonable request and can be accessed via the Zenodo link: https://zenodo.org/records/20687841 (accessed on 7 June 2026).
